# Applications of zeolite-zirconia-copper nanocomposites as a new asphaltene inhibitor for improving permeability reduction during CO_2_ flooding

**DOI:** 10.1038/s41598-022-09940-0

**Published:** 2022-04-13

**Authors:** Mohsen Mansouri, Yaser Ahmadi

**Affiliations:** Chemical and Petroleum Engineering Department, Ilam University, P.O. Box 69315/516, Ilam, Iran

**Keywords:** Chemistry, Chemical engineering

## Abstract

Using nanoparticles for adsorbing asphaltene was known as an efficient method among researchers for crude oil upgrading. In this study, zeolite-zirconia-copper nanocomposites (NCs) have been synthesized and characterized with Scanning electron microscopy (SEM), X-ray diffraction (XRD), Brunauer–Emmett–Teller (BET), and energy-dispersive X-ray (EDX). Then, CO_2_-oil interfacial tension (IFT) tests, Ultraviolet–visible spectroscopy (UV–Vis) Langmuir and Freundlich isotherm models, asphaltene precipitation tests at static phase, and dynamic CO_2_ flooding tests were performed in the presence of NCs and the results were compared with zeolite nanoparticles. Based on the characterization results, zirconia-copper particles were distributed at the surface of zeolite with total dimensions less than 30 nm, and the specific surface areas of the NCs (327.82 m^2^/g) was less than the pure zeolite (369.48 m^2^/g). It was seen that NCs had a greater asphaltene adsorption capacity and the application of decreasing asphaltene precipitation was higher in comparison to the zeolite nanoparticles. Accordingly, NCs were selected for performing dynamic CO_2_ tests and investigation of the permeability and porosity reduction parameters at obtained static condition. After adding NCs at the dynamic phase, asphaltene depositions that occured after CO_2_ injection was decreased and permeability/porosity reduction parameters were improved.

## Introduction

Asphaltene precipitation is one of the main concern during oil production, and changing thermodynamic conditions such as pressure, composition and temperature were known as important factors for asphaltene precipitation^1,2^. Asphaltenes are insoluble in paraffin (heptane and pentane) and soluble in aromatics (toluene)^3–5^. During oil production, problem occurs in the presence of asphaltene particles such as plugging due to precipitating of this solid phase in crude oil^6,7^. Researchers introduced many methods for solving asphaltene precipitation problems such as treatments with mechanical approaches or adding solvents and surfactants. Due to specific characteristics of nanoparticles, using nanoparticles especially nanocopmosites were introduced as an efficient method among different researchers^8–15^. The main parameter in their study is asphaltene adsorption on nanoparticles surface which depends on many factors such as carboxylic, pyrrolic, pyridinic existence in asphaltene, type and nanoparticles characteristics, and nanoparticles-asphaltene interactions^16–25^. Various nanoparticles such as zeolite and metal oxides and zirconia find applications in different fields such as catalysis and adsorption^26–31^. Zeolite is known as a reliable adsorbent of asphaltene, and hydrated aluminosilicate is the most important part of zeolite^26,27^, and these nanoparticles are used as support materials for stabilizing nanoparticles to produce high efficacy nanocomposites^32,33^. Zeolite nanoparticles were used for oil upgrading and cracking, and viscosity reduction^34^, and Kashefi et al.^35^ show that zeolite nanoparticles can be used as an efficient material for asphaltene adsorption and removing asphaltene deposition in the porous media. Furthermore, zirconia nanoparticles were known as an efficient materials for oil upgrading^36^, and showed excellent stability especially at high temperature ranges^37^.

Hosseinpour et al.^28^, shows that zirconia samples with surface acid sites have high BET specific surface area values compared to other nanoparticles such as Fe_2_O_3_, NiO, and WO_3_ which shows the strength of interactions between the asphaltenes and this sample. Other nanoparticles which are widely used due to their availability and low cost are copper oxide nanoparticles. Copper oxide nanoparticles have a wide variety of applications including efficient adsorbent^38–43^, foam stabilization^44^, EOR^45^, and environmental remediation^46^. Changing composition due to adding CO_2_ gas was known as a main concern in porous media, and it was found that asphaltene deposition especially at the inlet part of the core caused formation damage which should be fully considered^47–49^. Wang et al.^50^, investigated blockage degree during asphaltene precipitation during CO_2_ flooding, and based on the results, asphaltene precipitation which occurs in both large and small sizes correlated with permeability reduction. Moreover, it was found that the agglomeration problem relevant to nanoparticles can be solved by using nanocomposites^51^. In this study, zeolite-zirconia-copper oxide nanocopmosites (NCs) were used for surveying asphaltene adsorption on the surface and removal of asphaltene as an asphaltene inhibitor and the results compared with zeolite nanoparticles as a reference. As NCs had better results in the static phase and were selected for performing dynamic CO_2_ tests. Although the application of zeolite, zirconia, copper oxide at different cases were surveyed in previous studies such as asphaltene adsorption, introducing NCs as a possible asphaltene adsorbent and dynamic CO_2_ tests were not covered according to our knowledge. In first phase, adsorption behavior during CO_2_-oil IFT tests were observed and results was matched with real natural depletion tests in the presence of NCs and zeolite nanoparticles. Natural depletion was used as the main source of making asphaltene precipitation by changing pressures. The main points in natural depletion tests were selected based on the CO_2_-oil interfacial tension (IFT) tests (higher adsorption potential points). Two tests of CO_2_-oil interfacial tension (IFT) and Ultraviolet–visible spectroscopy (UV–Vis) spectrophotometer were used for describing asphaltene adsorption on the nanoparticles surface, and two different isotherm models of Langmuir and Freundlich were completely surveyed in the presence of NCs and zeolite nanoparticles. Finally, based on the static phase results, NCs were selected for performing dynamic CO_2_ tests and improving permeability reduction/porosity reduction parameters.

## Materials and methodology

### Materials

Crude oil was collected from one of the Iranian oil reservoirs in the west with an oil density of 0.864 g/cm^3^ and viscosity of 9.9 cP at 40 °C. Table 1 shows reservoir oil composition and the results of saturate-aromatic-resin-asphaltene (SARA).

**Table 1:** Reservoir oil composition and SARA tests.

Components	Reservoir oil (Mole %)	Components	Reservoir oil (Mole %)
N_2_	0.08	nC_4_	5.77
CO_2_	0.56	iC_5_	3.7
C_1_	3.44	nC_5_	5.3
C_2_	1.88	C_6_	5.92
C_3_	3.45	C_7+_	68.67

Saturate = 50.2%, Aromatic = 26.10%, Resin = 4.70%, Asphaltene = 22%.

Gas and brine permeability and porosity results and other specifications carbonate plug which were used in the dynamic CO_2_ test were shown in Table 2.

**Table 2:** Properties of plug for performing dynamic CO_2_ tests.

Properties	Carbonate plug
Length (cm)	4.80
Diameter (cm)	3.55
Gas pore volume (cc)	10.02
Gas permeability (cc)	8.43
Gas porosity (%)	17.30
Brine pore volume (cc)	11.10
Brine porosity (%)	19.47
Brine permeability (mD)	0.15

For extracting asphaltenes, the standard IP143 method was used^52^, and normal heptane 99%, toluene 99%, ethanol 99% and Watson paper grade 42 with a thickness of 0.22 µm were prepared from the Merck brand.

### Methodology

#### Synthesis of zeolite nanoparticles

For preparing zeolite, below 7 steps were performed:

1. Preparing the gel with the percentage molar of Al_2_O_3_: 46 SiO_2_: 2.7 TPA: 5 Na_2_O: 1.3 Trien: 2500 H_2_O.
2. Pouring the prepaid gel into a Teflon autoclave chamber (up to volume of 70%).
3. Placing autoclave in a temperature-controlled setup for 72 h.
4. Cool the autoclave and remove the products.
5. Washing the product with water (up to pH = 7).
6. Drying the resulting powder at 100 °C for 12 h.
7. Calcined the product at 550 °C for 8 h.

#### Synthesis of zeolite-zirconia-copper oxide nanocomposites

Below main steps were used for the synthesis of NCs.

1. Dissolving ZrCl4 in 50 ml propanol and obtaining Na-ZSM-5 zeolite gel.
2. Adding 5 ml (%30 v/v) H_2_O_2_ o the solution.
3. Setting the pH of zirconia gel solution at 9.
4. Preparing ZSM-5 with dissolving zeolite powder and Cu(NO_3_)_3_.6H_2_O in water
5. Setting the pH of green obtained solution at 9 with adding 10 ml NH_3_.
6. Obtaining NCs with adding ZSM-5:ZrO_2_:CuO = 70:25:5, respectively.
7. Stirring and aging of the obtained NCs for two days.
8. Performing filtration and calcination process at 300 °C for three hours.

#### Interfacial tension (IFT) tests

**Figure 1:**
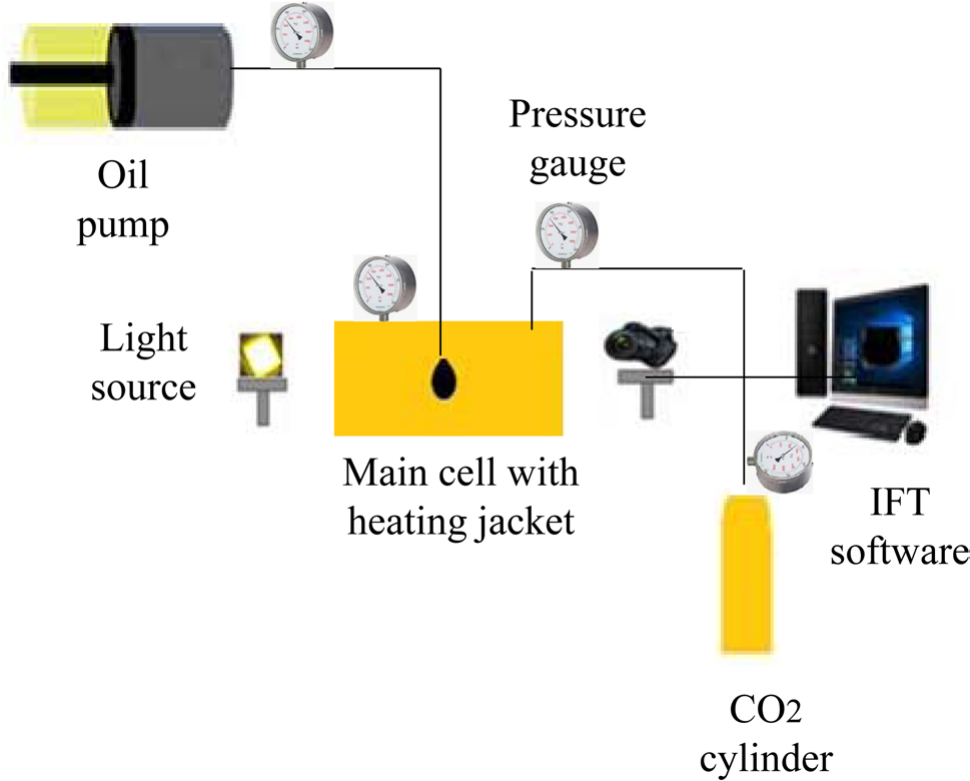
Schematic of high pressure and high temperature interfacial tension for CO_2_-oil tests.

CO_2_-Oil interfacial tension tests were performed with experimental setup as Fig. 1. This set up can measure interfacial tension at specific pressure and temperature.

IFT setup contained different parts such as high pressure dioxide carbon cylinder, high-pressure and high-temperature cells, pumps for injecting the crude oil, pressure gauges, temperature sensor, and data acquisition software. Four steps were used for obtaining CO_2_-oil IFT as:

1. Using Nitrogen for flushing the lines and main cell.
2. Injecting dioxide carbon gas through the main cell.
3. Pumped the oil (or oil with NCs) for obtaining oil droplets as shown in Fig. 1.
4. Using software for IFT measurements.

#### Natural depletion tests

**Figure 2:**
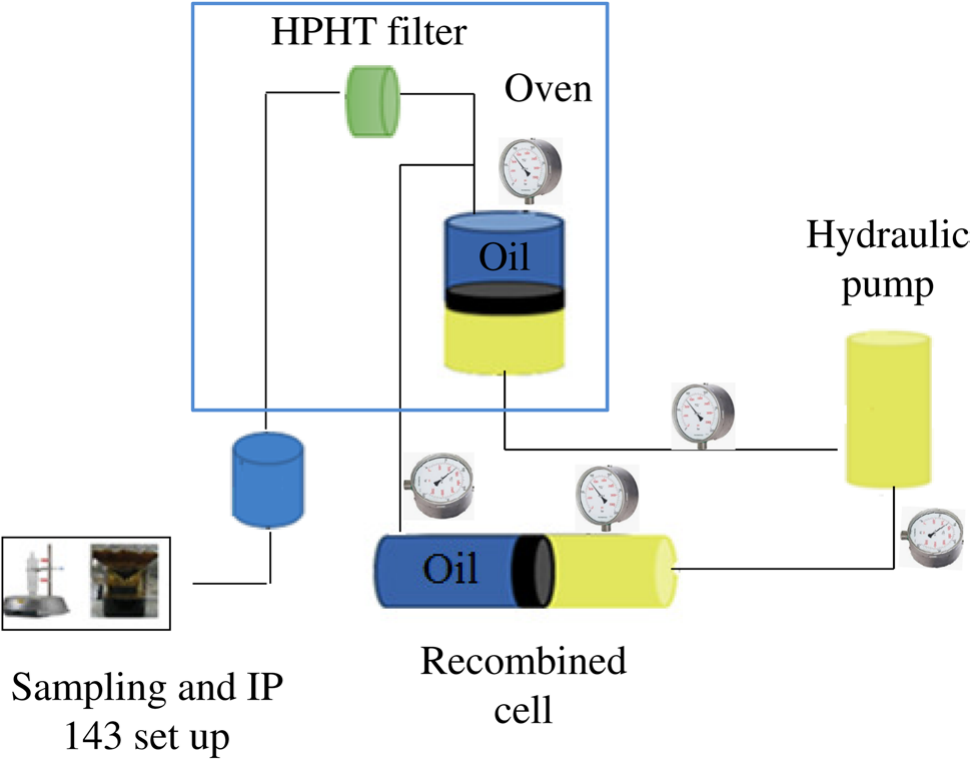
Set up for performing natural depletion.

Natural depletion tests were performed with static apparatus as shown in Fig. 2, and it was used high pressure metal filter with 0.22 micron size. The set up was contained a pump for injecting hydraulic fluid below main piston, high pressure and temperature recombined cell, main cell, high pressure 0.22 micron metal filter, cell of fluid sampling, gauges for detecting pressure, and temperature sensor. Below five steps were used for performing natural depletion at static phase:

1. Transferring high pressure crude oil sample through main cell.
2. Stirring the main cell for 24 h.
3. Opening outlet needle valve and pass the high-pressure sample through 0.22 micron metal filter.
4. Obtaining asphaltene content percent with standard IP143 method.
5. Asphaltene precipitation Calculation with subtracting asphaltene content from step 4 from asphaltene content at the initial case.

#### Batch adsorption experiment

Surveying adsorption of asphaltene on NCs or zeolite nanoparticles surface was performed with batch adsorption tests with toluene solution. Below five steps were used during this adsorption test:

1. Adding certain amounts of NCs or zeolite’s nanoparticles concentration.
2. Shaking solution at 200 rpm for 4 hours^35^.
3. Using centrifuge at 3000 ppm and 30 min for separating asphaltene which adsorbed on NCs or zeolite nanoparticles surface.
4. Measuring remaining asphaltene concentration.
5. Calculating the amount of adsorbed asphaltene on nanoparticles surface with Eq. 1 as below:
  1 $$\mathrm{Q}=\frac{({\mathrm{C}}_{\mathrm{o}}-{\mathrm{C}}_{\mathrm{e}})}{\mathrm{m}}\boldsymbol{*}\mathrm{V}$$

The main items in Eq. 1 and the relevant units are Initial asphaltene concentration ($${C}_{o})$$, mg/L, asphaltene equilibrium concentration $${(C}_{e})$$, mg/L, volume of solution (V), L, NCs mass (m), mg.

#### Dynamic experimental CO_2_ procedure

**Figure 3:**
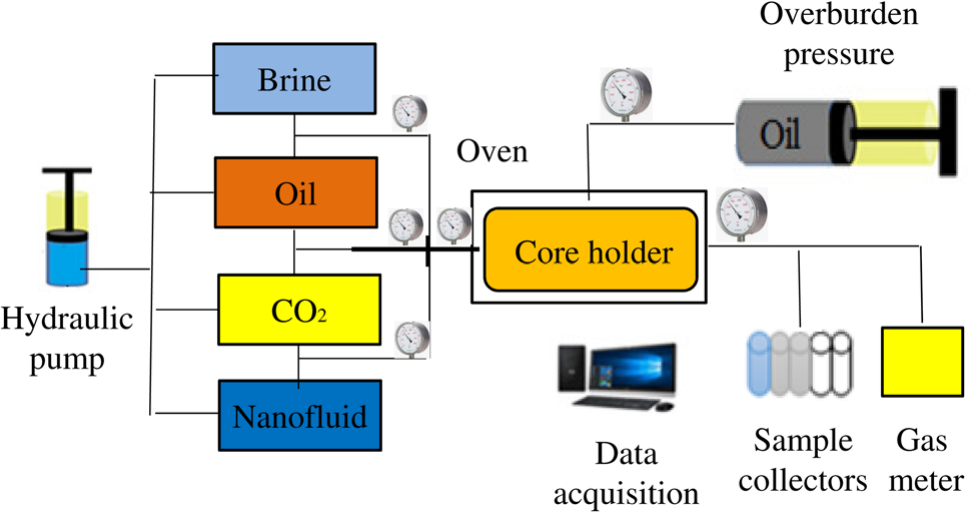
Dynamic apparatus for performing dynamic CO_2_ tests.

A dynamic apparatus schematic of setup that used to perform dynamic CO_2_ tests is shown in Fig. 3.

The dynamic set up was contained three different vessels of brine, nanofluids, and crude oil, hydraulic pump, core holder for holding carbonate core plug, pressure and temperature sensors, overburden pump, DP sensors, and acquisition data. Below five steps were used for performing dynamic CO_2_ tests in the presence of NCs:

1. Injecting formation water (0.1 cc/min) and calculate water permeability
2. Obtaining residual water saturation with performing oil injection
3. Calculation of effective oil permeability at residual water saturation
4. Injecting simultaneous oil and CO_2_
5. Obtaining permeability reduction

For nanocopmosites, the procedure was the same except for step 4 inwhich oil contains NCs 30 ppm at 1700 Psi and 40 °C. Figure 5S show the experimental approach which was used in this study:

## Results and discussion

### Characterization

**Figure 4:**
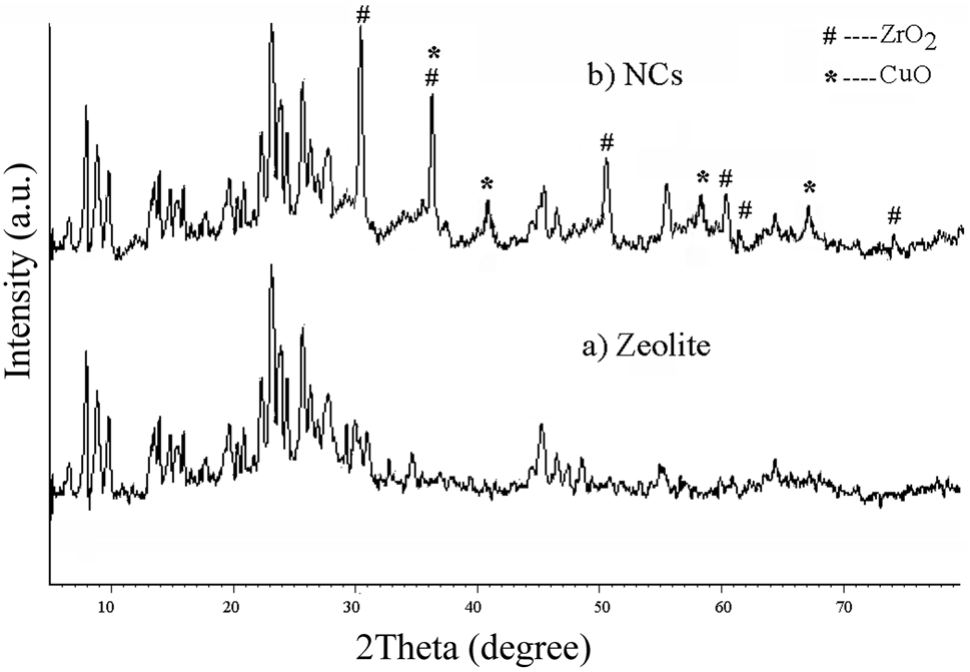
XRD pattern of (**a**) zeolite and (**b**) zeolite-zirconia-copper nanocomposites (NCs).

Figure 4a, b shows X-ray analysis for detecting the structure of zeolite and NCs nanoparticles, respectively. Zeolite structure is determined through d_hkl_ = 7.5°, 8.5°, 23° and 24° reflections^53^. Figure 4b shows zirconia planes based on the JCPDS 79-1771 ([2θ; planes] are [30.47, 36.78°, 50.17, 60.23, 61.81 and 73.59; 111, 200, 220, 311, 222 and 400]. Moreover, Copper oxide particles were detected according to Zhu et al. group (2θ = 36.12°, 40.82°, 58.61° and 67.38)^54^. This shows the formation of CuO and ZrO_2_ nanoparticles over the formed zeolite framework.

Equation 2 was used for determining nanoparticles size:

2 $$ \tau = \frac{k\,\lambda }{{\beta \,\cos \theta }} $$

τ, *k,* λ, *β,* and *θ* are crystalline size (nm), shape factor (0.9), x-rays wavelength (0.154 nm), line broadening, and Bragg angle. NCs calculated average size was 30.21 nm.

**Figure 5:**
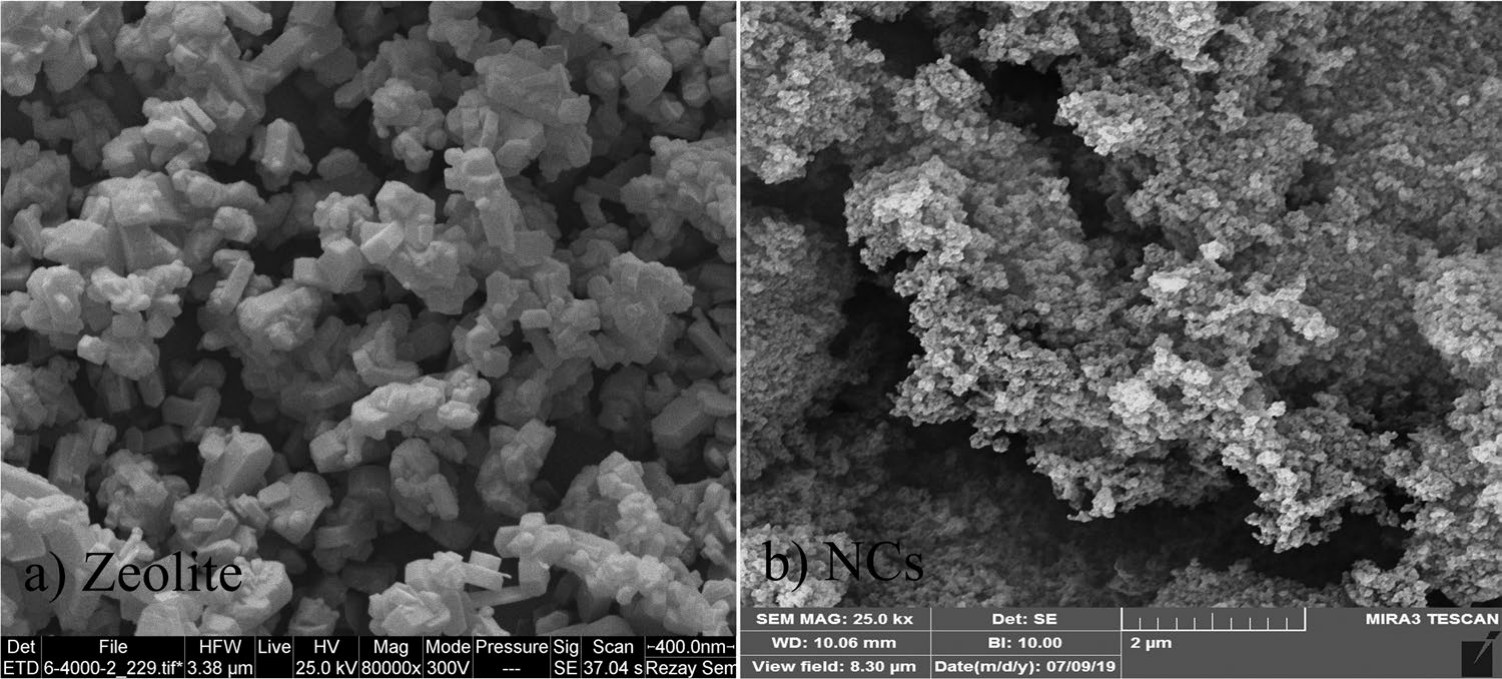
SEM images of a: zeolite and b: zeolite-zirconia-copper nanocomposites.

The morphological surface and size of nanoparticles were investigated by SEM. Images obtained from nano zeolite and synthesized zeolite-zirconia-copper nanocomposites (NCs) are shown in Fig. 5. From a morphological point of view as shown in Fig. 5a, the zeolite sample is composed of a large number of coffin-shaped units with relatively similar dimensions of about 50 nm. In this image, it is possible to see the uneven surfaces of the bed and cavities, which will increase the specific surface area and adsorption capacity.About gained SEM image from zeolite-zirconia-copper nanocomposites (NCs) as Fig. 5b, it can be observed that the zirconia-copper particles were uniformly distributed at surface of zeolite with dimensions less than 30 nm. This is a sign of the successful stabilization of nanoparticles on the zeolite substrate.

The surface area, pore size, and pore volume of the pure zeolite and zeolite-zirconia-copper nanocomposites (NCs) were measured by BET. The results are summarized in Table 3. The specific surface areas of the NCs (327.82 m^2^/g) are less than the pure zeolite (369.48 m^2^/g) as a result of introducing of ZrO_2_ and CuO. An increase in the ZrO_2_ and CuO contents decreases the specific surface area due to increasing the crystal size and the pore blockage of the support. Compared to NCs, pure zeolite has a higher specific surface area and higher pore volume, but a lower average pore diameter.

**Table 3:** Textural properties of the prepared materials.

Sample	Surface area (m^2^/g)	Pore volume (cm^3^/g)	Average pore diameter (nm)
zeolite	369.5	0.12	1.02
NCs	327.8	0.11	1.22

NCs = zeolite-zirconia-copper nanocomposites.

**Figure 6:**
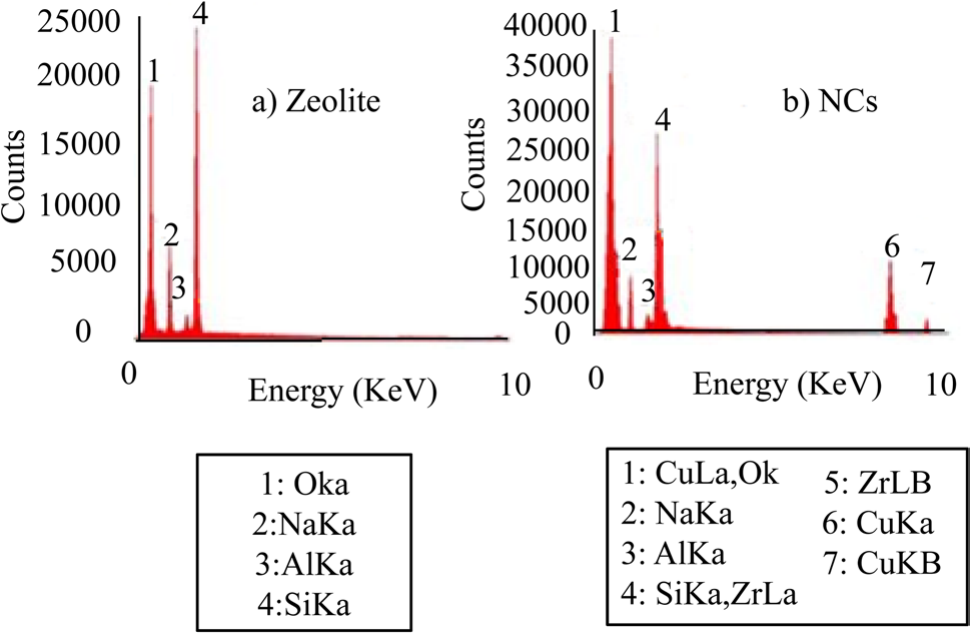
EDX images of (**a**) zeolite (left) and (**b**) zeolite-zirconia*-*copper nanocomposites (right).

Figure 6 and Table 4 show EDX results for zeolite nanoparticles and NCs. Figure 6a shows different elements in the zeolite nanoparticles such as Si (31.49), Al (3.38), Na (16.32) and O (48.81)^55^. Different elements of Al, Si, O, and Na were observed in the NCs composition as Fig. 6b and Table 4. Zirconia and copper were observed in zeolite nanoparticles based on EDX images.

**Table 4:** EDX data for zeolite and NCs.

Elements	Atomic percentage (At%)	
	Zeolite	NCs
Si	31.49	19.89
Al	3.38	2.44
Na	16.32	6.84
O	48.81	54.91
Zr	–	13.21
Cu	–	4.18

### Asphaltene adsorption isotherms in the presence of NCs and zeolite nanoparticles

Langmuir and Freundlich isotherm models which were used in this study are expressed in Eqs. 3 and 4^56^.

3 $$ Q_{e} = Q_{m} \frac{{K_{L} C_{e} }}{{1 + K_{L} C_{e} }} $$

4 $$ Q_{e} = K_{F} C_{e}^{1/n} $$

Equations 5 and 6 are expressed in linear form as:

5 $$ \frac{{C_{e} }}{{Q_{e} }} = \frac{1}{{Q_{m} K_{L} }} + \frac{{C_{e} }}{{Q_{m} }} $$

6 $$ {\text{Ln}}\left( {Q_{e} } \right) = {\text{Ln}}\left( {K_{F} } \right) + \frac{1}{n}{\text{Ln}}\left( {C_{e} } \right) $$

**Figure 7:**
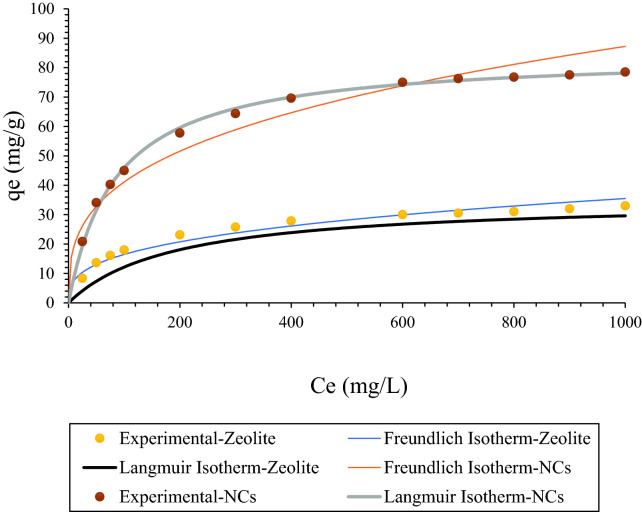
Asphaltene adsorption models (Langmuir and Freundlich) for NCs and zeolite nanoparticles.

Asphaltene adsorption on NCs and zeolite’s surface (mg/g), equilibrium concentration of NCs and zeolite (mg/L), maximum asphaltene adsorption per grams of NCs or zeolite (mg/g), Langmuir constant of adsorption, the capacity of adsorption in Freundlich isotherm ([mg/g] [L/mg]), and intensity factor in Freundlich isotherm were shown with Q_e_, Ce, Q_m,_ K_L_, K_F_, and 1/n, respectively. Figure 7 shows asphaltene adsorption on the NCs nanocomposites and zeolite’s surface in the batch experiment tests up to 1000 ppm. As it is clear from the graph, there is two different slopes below 100 ppm and after that. At first slope (below 100 ppm), the slope was dramatically increased. Then, asphaltene adsorption on the NCs nanocomposites and zeolite’s surfaces were reached to around 78.5 mg/g (0.213 mg/m^2^), and 33 mg/g (0.101 mg/m^2^), respectively. Accordingly NCs were adsorbed more asphaltene on its surface compared to zeolite nanoparticles. It was concluded from previous research that the type of sorbent is affected on the amount and type of adsorption^19,57,58^. According to the isotherm model results in this study, the adsorption data was adapted well with Langmuir isotherm than Freundlich isotherm, and this shows that adsorption surface was monolayer and homogeneous. Freundlich isotherm occurs in the surface that has different energy with the heterogeneous surface^58^. From the comparison between Freundlich isotherm between zeolite and NCs, it was observed that the data less adopted with NCs in comparison zeolite. Langmuir and Freundlich isotherm linear plots are shown in Figs. 2S and 3S, respectively. All experiments in this study were replicated three times with the maximum uncertainty of ± 5%.

Isotherm parameters of both isotherm models were shown in Table 5. According to the results, NCs have better adsorption capacity than zeolite nanoparticles. The maximum adsorption ratio of NCs to the zeolite (qm NCs/qm zeolite) was 2.41. Furthermore, NCs had higher adsorption capacity in comparison zeolite, and the KF NCs / K_F_ zeolite was 2.5782. Baninaam et al. was investigated the isothermal behavior of ZSM-5, and based on their results, q_m_ was 21.7024 mg/g which is less than both nanoparticles in this study^59^.

**Table 5:** Langmuir and Freundlich isotherm model constants.

Nanoparticles	Langmuir			Freundlich		
	q_m_ [mg/g]	K_L_ [L/mg]	R^2^	1/n	K_F_ [mg/g][L/mg]	R^2^
NCs	84.7458	0.01181	0.9997	0.3265	9.1437	0.9411
zeolite	35.2113	0.01052	0.9984	0.3334	3.5465	0.9504

### Nanoparticles adsorption before and after asphaltene adsorption

**Figure 8:**
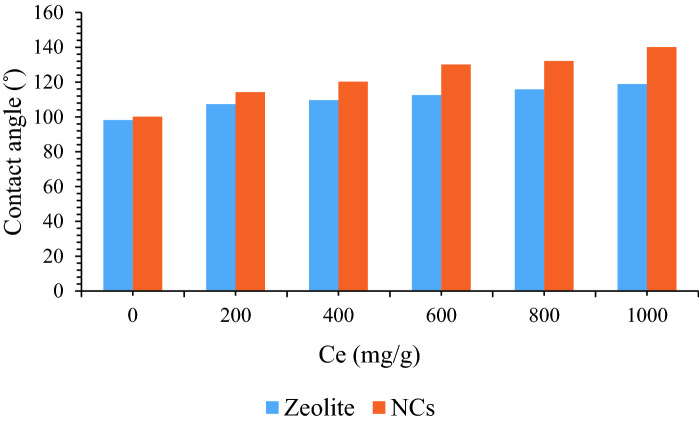
Contact angle before and after asphaltene adsorption at different asphaltene concentration.

To have better understanding of the behavior of both NCs and zeolite nanoparticles, contact angle were performed at different equilibrium asphaltene concentrations includes 200, 400, 600, 800, and 1000 mg/L. At each asphaltene solution, nanoparticles surfaces were immersed in for 48 h, and the contact angles were measured in the presence of water^60^. Figure 8 shows asphaltene adsorption before and after asphaltene adsorption. In the presence of NCs, contact angles had higher results in comparison to the zeolite which were in a good agreement with batch adsorption experiment (Fig. 7). In other words, as NCs had more contact angle due to adsorb more asphaltene on its surface in comparison to zeolite.

### Effect of nanoparticles concentration and pressure on CO_2_-crude oil interfacial tension

**Figure 9:**
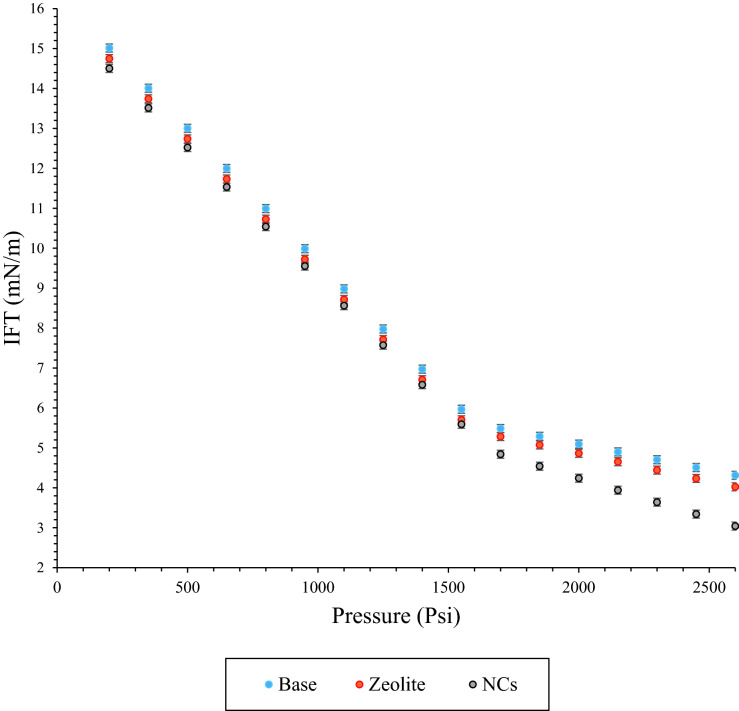
Effects of pressure and nanoparticles (NCs and zeolite) on CO_2_-oil IFT.

The effects of NCs and zeolite nanoparticles on the CO_2_-Oil IFT was shown in Fig. 9. As can be seen from Figure, two main slopes were observed with/without nanoparticles. The slope of second region was decreased at high pressures. In other words, at high pressure asphaltene adsorbed on the nanoparticles surface and have not tend to aggregated in compared to first region and low pressure. Slope in the second region was increased but not as much as first region which shows that nanoparticles reduced asphaltene precipitation. Other main point is that, although nanoparticles decreased asphaltene precipitation but it did not stop it completely^61^. According to the results, there are two different ranges from 200 to 2600 Psi. Interfacial tension of CO_2_-Oil was decreased due to dissolving CO_2_ in the crude oil. The slope in this graph was changed due to forming the aggregate of asphaltene particles. According to Fig. 9, NCs were changed the slope in the second region more than Na-ZSM-5 zeolite, and in other words adsorbed higher amounts of asphaltene on its surface. As it was mentioned in the previous section, NCs had the better asphaltene adoption in comparison to zeolite nanoparticles which confirmed these results. Moreover, one of the other essential results from the results was delaying agglomeration in the presence of NCs and zeolite nanoparticles, and it was confirmed that NCs had better results than zeolite. Previously Kazemzadeh et al. was seen the same results in the presence of Fe_3_O_4_ nanoparticles^61^. Based on their results, the main mechanism of IFT reduction in the presence of nanocopmosites and zeolite nanoparticle were asphaltene adsorption onto the surface of the nanocomposites.

Seven pressures from the second region (1700–2600 Psi) were selected for obtaining the relation between these high adsorber points with adsorption in the real crude oil in natural depletion tests. As it was mentioned, there are two different slopes of CO_2_-Oil in the base and both nanoparticles of NCs and zeolite, and Table 6 summarized different equations in these two regions. 2nd to 1st slope ratio was 19.403% at the base case, and 2nd to 1st ratio increased to 20.895% and 30.303% in the presence of NCs and zeolite at 30 ppm, respectively. Thus, NCs had better performance with regards adsorbing asphaltene on its surface in comparison zeolite.

**Table 6:** Changes in CO_2_-oil IFT slope ratio in the presence of NCs and zeolite nanoparticles.

Nanoparticles type	Nanoparticle concentration (ppm)	Region	Equation IFT (mN/m)	Ratio of the IFT slope in 2nd to the 1st region (%)
Base	–	1:(200–1550)Psi	IFT = -0.0067P + 16.088	19.403
		2:(1700–2600)Psi	IFT = -0.0013P + 7.6943	
Zeolite nanoparticles	30	1:(200–1550)Psi	IFT = -0.0067P + 16.353	20.895
		2:(1700–2600)Psi	IFT = -0.0014P + 7.6647	
NCs	30	1:(200–1550)Psi	IFT = -0.0066P + 15.823	30.303
		2:(1700–2600)Psi	IFT = -0.0020P + 8.2414	

### Natural depletion tests in the presence of nanoparticles

**Figure 10:**
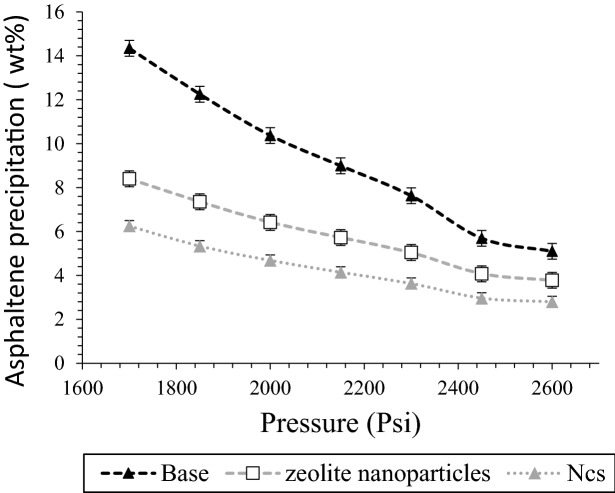
Effects of NCs and zeolite nanoparticles on asphaltene precipitation during natural depletion tests.

As it was mentioned in the previous section, seven pressure of 1700, 1850, 2000, 2150, 2300, 2450, and 2600 Psi have selected as high adsorption points for static phase and performing natural depletion tests in the presence of NCs and zeolite nanoparticles. Asphaltene precipitation content versus pressures at static pressure were shown in Fig. 10, and effects of both NCs and zeolite nanoparticles were observed according to this graph. It was seen asphaltene precipitation of NCs and zeolite nanoparticles decreased from (5.10 wt%, 14.34 wt%) to (3.78 wt%, 8.40 wt%) and (2.8 wt%, 6.25 wt%), respectively during pressure reduction from 2600 to 1700 Psi. Accordingly, based on the results, NCs had better results for decreasing asphaltene precipitation in the static phase in comparison to zeolite, and it can be in a direct relation with higher adsorption potential in the previous steps.

### Dynamic CO_2_ tests in the presence of NCs

According to the static phase results, NCs had better results than zeolite in aspect of asphaltene adsorption and asphaltene precipitation reduction. Thus it was selected for surveying permeability/porosity reduction parameters. As it is clear from the Table 7, NCs had better results in lower pressure for asphaltene precipitation at 1700 Psi, 40 °C, and 30 ppm were selected for performing CO_2_ flooding at dynamic phase.

**Table 7:** Difference between Initial asphaltene precipitation and asphaltene precipitation in the presence of NCs.

Pressure	Initial asphaltene precipitation	Asphaltene precipitation in the presence of NCs	(Initial-NCs) asphaltene precipitation
Psi	wt%	wt%	wt%
1700	14.34	6.25	8.09
1850	12.25	5.33	6.92
2000	10.37	4.68	5.69
2150	8.99	4.14	4.85
2300	7.63	3.63	4.00
2450	5.69	2.96	2.73
2600	5.10	2.80	2.30

**Figure 11:**
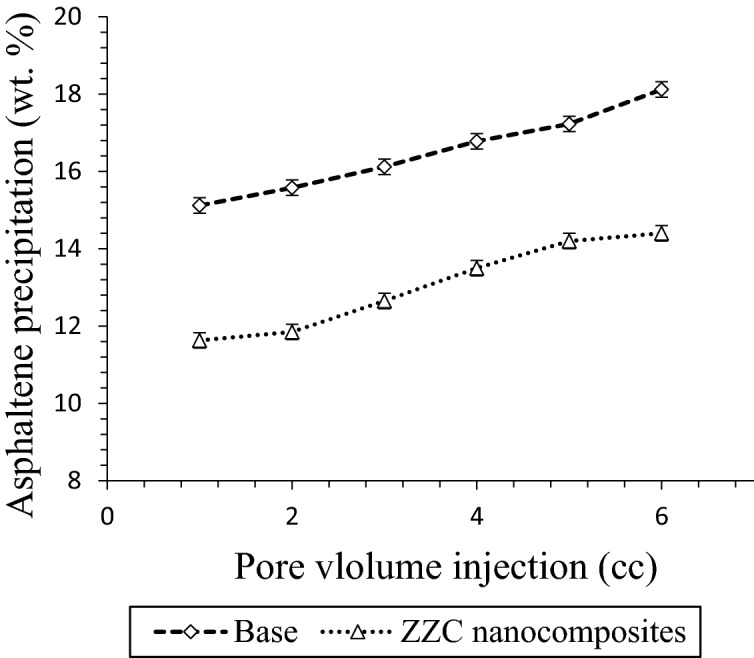
Effect of NCs on the asphaltene deposition during CO_2_ flooding.

One of the essential factors in porous media is considering deposition condition. As it was shown in the static phase, NCs had better performance in comparison to zeolite. Accordingly, it was selected for surveying its effect on the deposition rate in porous media. Dynamic displacement tests were used for CO_2_ flooding in the presence of NCs. As it is clear from Fig. 11, asphaltene deposition in the presence of gas was increased during CO_2_ flooding. By measuring the asphaltene content of injected and produced oil samples, one can estimate the amount of deposition in porous media. The asphaltene of produced oil was measured by the IP143 standard technique. After adding NCs in crude oil, asphaltene precipitation was reduced. As CO_2_ gas volume was increased from 1 to 6 pore volume, asphaltene precipitation (wt%) was decreased from (15.12, 18.12) to (11.85, 14.44), respectively.

**Figure 12:**
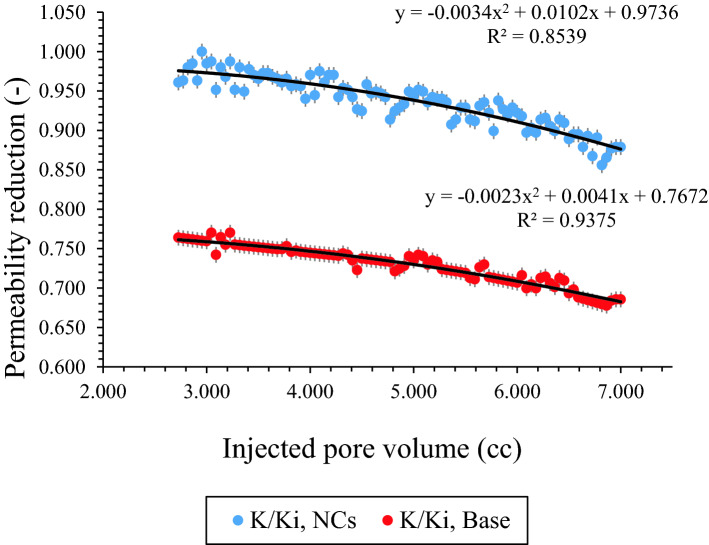
Permeability reduction in the presence of NCs during CO_2_ flooding.

**Figure 13:**
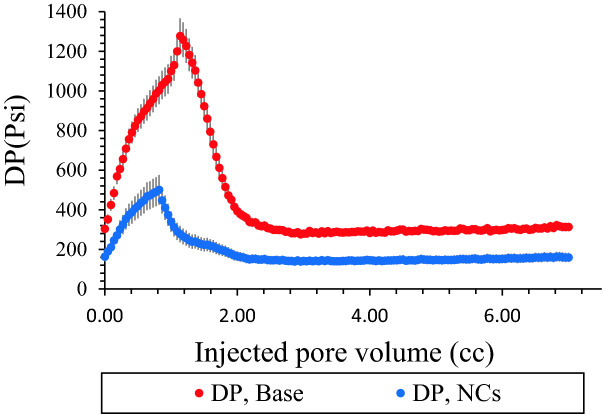
Differential pressure in the presence of NCs during CO_2_ flooding.

As it was mentioned before, after saturation of carbonate core sample with brine, recombined oil and CO_2_ [25 Mole % which was more than initial onset value (20 Mole %) at static phase] displaced through the low permeability carbonate core and initial permeability reduction was recorded. Then, same procedure was performed in the presence of NCs [dispersed in oil] at 30 ppm, 1700 Psi, and 40 °C as Figs. 12 and 13. Many other cases and laboratory studies have been reported on precipitation and deposition of asphaltenes in porous media during immiscible or miscible gas flooding operations^62–67^. Figure 12 shows permeability reduction in the presence of NCs, and based on the results, NCs improved permeability reduction in porous media. Figure 13 shows differential pressure between inlet and outlet cores for both tests. As it is clear from the graph, most pressure drop was occurs at initial part which asphaltene deposition problem was severe at this point. Moreover, NCs decreased pressure drop successfully in comparison to base CO_2_ flooding. The precipitated asphaltenes left in the reservoir core plugs reaches its lowest value. An explanation for this phenomenon is that the total density of solution increase due to adding NCs, and solubility parameter of the mixture increase within the presence of NCs. Hence, the oil becomes stable and the asphaltenes precipitation decreases^68^.

**Figure 14:**
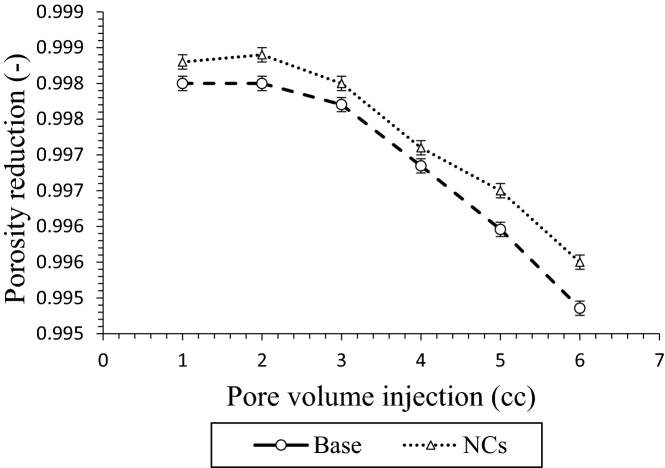
Porosity reduction in the presence of NCs during CO_2_ flooding.

There is direct relation between porosity reduction and asphaltene deposition rate. Figure 14 shows the porosity variations of low permeability carbonate reservoir versus the amounts of pore volume. As it was observed in the previous steps, NCs was decreased asphaltene precipitation in static phase at 30 ppm, and at same concentration asphaltene deposition was decreased in carbonate reservoir. Moreover, higher porosity reduction was seen at higher asphaltene deposition points.

## Conclusions

Zeolite-zirconia-copper oxide nanocomposites (NCs) have been synthesized successfully with average size of 30 nm and it was used for asphaltene adsorption and solving asphaltene precipitation problems and the results compared with zeolite nanoparticles. Results show that NCs nanoparticles adsorbed higher amount of asphaltene and asphaltene precipitation decreased more in the presence of NCs nanoparticles than zeolite nanoparticles which reveal this point that these nanocomposites can be used efficiently as an asphaltene inhibitor and the agglomeration process was delayed efficiently. Adsorption data fitted well with the Langmuir model compared to the Freundlich model, which shows that the adsorption occurs on a homogeneous surface with monolayer coverage in the presence of both nanoparticles. Based on the BET results, NCs has lower surface area, higher pore volume but higher diameter in comparison to zeolite nanoparticles. EDX analysis confirmed that NCs synthesized successfully. There is two different slope in CO_2_-oil IFT readings as pressure increases (200 Psi-2600 Psi), and the second slope is slower than the first one which is due to aggregation of asphaltene. Seven pressures of 1700, 1850, 2000, 2150, 2300, 2450, 2600 Psi and NCs and zeolite nanoparticles at a concentration of 30 ppm were selected for performing natural depletion tests. Itwas concluded that as the pressure decreased from (2600 Psi to 1700 Psi), asphaltene precipitation in the presence of NCs and zeolite decreased from (Base: 5.10 wt%, 14.24 wt%) to (2.80 wt%, 6.25 wt%) and (3.78 wt%, 8.40 wt%), respectively. It was observed that the NCs has higher efficiency with regards adsorbing asphaltene on its surface and decreasing asphaltene precipitation during natural depletion in comparison to zeolite nanoparticles, and it was selected for performing CO_2_ dynamic tests. In dynamic phase. It was observed that NCs had high potential material for improving permeability impairment, porosity reduction, and asphaltene deposition rate during CO_2_ flooding tests.

## Supplementary Information

Supplementary Information.
