# Supplementary material for: Applications of zeolite-zirconia-copper nanocomposites as a new asphaltene inhibitor for improving permeability reduction during CO2 flooding
Source: Sci Rep. 2022 Apr 13;12:6209. doi: 10.1038/s41598-022-09940-0 (PMC9007961; doi:10.1038/s41598-022-09940-0)
Supplement: Supplementary file 1 — Supplementary Information. [file 41598_2022_9940_MOESM1_ESM.docx]

Applications of Zeolite-Zirconia-[Copper](https://byjus.com/chemistry/copper-oxide/) Nanocomposites as a New Asphaltene Inhibitor for Improving Permeability Reduction during CO_2_ Flooding

Mohsen Mansouri^1^, Yaser Ahmadi^1*^

^1^ Chemical and Petroleum Engineering Department, Ilam University, P.O. Box 69315/516, Ilam, Iran


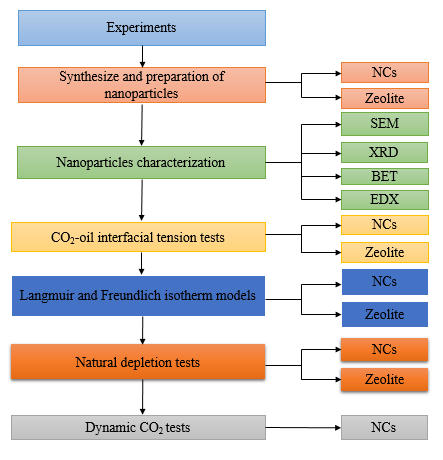


Figure 1S. Schematic of experimental procedures.

Figure 2S. Langmuir model constants for NCs and zeolite nanoparticles.

Figure 3S. Freundlich model constants for NCs and zeolite nanoparticles.
